# Decitabine combined with RDHAP regimen in relapsed/refractory diffuse large B cell lymphoma

**DOI:** 10.1002/cam4.5615

**Published:** 2023-01-25

**Authors:** Xiaoshuang Kong, Xudong Zhang, Mengjie Ding, Xiaoyan Feng, Meng Dong, Lei Zhang, Xiaorui Fu, Ling Li, Xin Li, Zhenchang Sun, Jiaqin Yan, Xinhua Wang, Xiaolong Wu, Qingjiang Chen, Mingzhi Zhang, Linan Zhu

**Affiliations:** ^1^ Department of Oncology Lymphoma Diagnosis and Treatment Centre of Henan Province, The First Affiliated Hospital of Zhengzhou University Zhengzhou Henan China

**Keywords:** chemotherapy, decitabine, diffuse large B‐cell lymphoma

## Abstract

**Background:**

There is an urgent need for effective treatment of patients with relapsed/refractory diffuse large B‐cell lymphoma (R/R‐DLBCL). This trial investigated the efficacy of decitabine in combination with rituximab, cisplatin, cytarabine, dexamethasone (RDHAP) in R/R‐DLBCL.

**Methods:**

56 patients were divided into two groups (decitabine‐RDHAP group. *n* = 35; RDHAP group, *n* = 21). The primary endpoints were the overall response rate (ORR) and duration of remission (DOR). Secondary objectives were toxicity, progression‐free survival (PFS), and overall survival (OS).

**Results:**

The ORR was 40% and 33% for decitabine‐RDHAP and RDHAP groups, respectively, with no difference between the groups. The DOR for the decitabine‐RDHAP regimen was higher than that for the RDHAP regimen (*p* = 0.044). After a median follow‐up of 12.0 months, the median PFS and OS were 7.0 and 17.0 months for in the decitabine‐RDHAP group and 5.0 and 9.0 months in the RDHAP group with no significant differences between the two groups (*p* = 0.47, 0.17). The incidence of adverse events was not significantly different between groups.

**Conclusion:**

The decitabine‐RDHAP regimen is effective and well tolerated, and is a promising salvage regimen for R/R‐DLBCL.

## Graphical abstract

A randomized phase II study of decitabine in combination withrituximab, cisplatin, cytarabine, dexamethasone (RDHAP) in relapsed/refractory diffuse large B cell lymphoma.

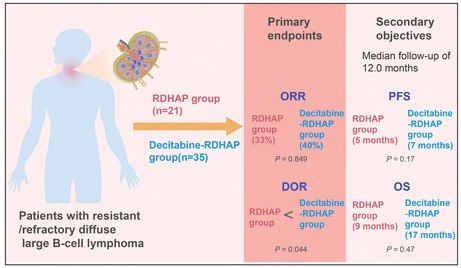

## 1 INTRODUCTION

Diffuse large B‐cell lymphoma (DLBCL) is the most common subtype of non‐Hodgkin lymphoma (NHL), accounting for approximately 30%–40% of cases. The current standard treatment is the R‐CHOP regimen, although 30%–40% of patients relapse or develop refractory diseases.^1^ Only 13% of these patients undergo high‐dose chemotherapy followed by autologous stem cell transplantation (HDT‐ASCT).^2^ Patients with relapsed/refractory (R/R) DLBCL who are not eligible for transplantation have limited treatment options and a poor prognosis, with a median survival (overall survival [OS]) of approximately 6 months.^3^ The National Comprehensive Cancer Network guidelines indicate the preferred clinical trials for patients with DLBCL who are not suitable for HDT‐ASCT.^4^ Therefore, novel drugs are urgently required to improve the survival of patients with R/R‐DLBCL.

Recently, abnormal DNA methylation was found to be related to the development and chemoresistance of lymphoma.^5^, ^6^ Promising outcomes have been reported with decitabine, a phase‐S DNA methylation inhibitor in hematological and solid tumor malignancies.^7^, ^8^, ^9^, ^10^, ^11^ Low‐dose decitabine can induce DNA demethylation and hematopoietic stem cell differentiation. Furthermore, decitabine was found to restore chemotherapy sensitivity to anthracyclines by reactivating SMAD1 expression.^12^, ^13^ Decitabine also has synergistic antitumor effects with cisplatin and cytarabine.^8^, ^14^ We conducted a prospective clinical experiment to determine the efficacy and safety of decitabine combined with the RDHAP (rituximab, dexamethasone, cytarabine, and cisplatin) in patients with R/R‐DLBCL.

## 2 METHODS

### 2.1 Patient study inclusion criteria

This was an investigator‐initiated, prospective, randomized, controlled, open‐label clinical trial of R/R‐DLBCL patients registered at www.clinicaltrials.gov (identifier: NCT03579082).

Patients were recruited for the study according to the following inclusion criteria: (1) age 14–70 years; (2) histologically confirmed DLBCL; (3) previous cyclophosphamide, doxorubicin, vincristine, and prednisone (CHOP) or rituximab plus CHOP (RCHOP) or failure of multiline treatments but not chemotherapy with the rituximab plus DHAP (RDHAP) regimen; (4) estimated survival time >3 months; (5) cases deemed unsuitable for transplantation, for reasons including disease progression, age, comorbidities, poor response to previous treatment, previous transplantation failure, and other conditions; (6) no chemotherapy contraindications; (7) at least one measurable lesion; (8) no uncontrolled medical disease. Relapsed DLBCL:complete response (CR) to initial chemotherapy was achieved and relapsed after 6 months. Refractory DLBCL: (1) tumor shrinkage <50% or disease progression after four cycles of standardized chemotherapy, (2) CR to initial chemotherapy was achieved and relapsed within 6 months, and (3) relapse after transplantation (refractory DLBCL can be diagnosed if one of these parameters is met).

Between August 2018 and December 2021, 56 patients (35 in the decitabine‐RDHAP group and 21 in the RDHAP group) were evaluated to assess treatment efficacy. The study was approved by the Human Ethics Committee of the First Affiliated Hospital of Zhengzhou University and was conducted in accordance with the principles outlined in the Declaration of Helsinki. All the patients or their family members signed an informed consent form.

### 2.2 Randomization

Patients were randomized by 1:1 assignment to either the decitabine‐RDHAP group or the RDHAP group using computer‐generated randomization lists.

Some patients, particularly those in the RDHAP group, discontinued the treatment or were lost to follow‐up due to economic distress, poor physical condition, poor tolerance to chemotherapy, the COVID‐19 pandemic, and rainstorm disaster in Henan in 2021, and finally, these patients were excluded from the study.

### 2.3 Chemotherapy

In the decitabine‐RDHAP group, decitabine was administered intravenously for 5 days at 10 mg/day, followed by a modified DHAP regimen. The DHAP regimen was modified because the enrolled patients were almost all in the advanced stage and bone marrow ability of patients was poor. The following RDHAP regimen was administered. Rituximab was then administered intravenously at 375 mg/m^2^ (in accordance with the standard rate infusion escalation protocol) in each cycle. Cisplatin was administered intravenously at a dose of 100 mg/m^2^, equally divided and administered on days 1–3. Cytarabine was administered intravenously at 2 g/m^2^ every 12 h (Q12H) on day 2, and 40 mg/day dexamethasone was administered on days 1–4. Cycles of decitabine‐RDHAP and the RDHAP regimens were repeated every 21 days for a maximum of six cycles.

### 2.4 Follow‐up

Treatment was discontinued in the following cases: (1) Imaging showed progressive disease (PD) requiring alternative treatment. (2) Patients were eligible for transplantation and requested to discontinue treatment and then underwent transplantation. (3) Patients themselves requested to withdraw from the clinical trial or withdrawal was considered medically necessary by the investigators.

Chemotherapy with cisplatin and cytarabine was reduced by 20% (rituximab, decitabine, and dexamethasone did not require reduction) if patients experienced grade 3–4 adverse events (AEs) that did not resolve within 2 weeks.

Recombinant human granulocyte colony‐stimulating factor and recombinant human thrombopoietin were administered to patients who developed neutropenia and thrombocytopenia as supportive therapy or secondary prevention in the next cycle.

### 2.5 Endpoints

The primary endpoints were objective response rate (ORR) and duration of response (DOR), and the secondary endpoints were toxicity, progression‐free survival (PFS), and OS.

### 2.6 Statistical analysis

Patient characteristics and AEs were compared between the decitabine‐RDHAP and RDHAP group using the χ^2^ test or Fisher's exact test for discrete variables and the Wilcoxon Mann–Whitney test for continuous variables. The response rates of the decitabine‐RDHAP and RDHAP groups were compared by using the Wilcoxon (Mann–Whitney) rank‐sum test. OS, PFS, DOR, and duration of disease control (DDC) were estimated by using the Kaplan–Meier method. Survival analysis of patient subgroups was compared using a meta‐analysis. *p* < 0.05 indicated a statistically significant difference. Statistical analyses were performed using Prism version 8.0.2 for Windows (GraphPad Software).

### 2.7 Evaluation criteria

Positron emission tomography‐computed tomography (PET‐CT) or computed tomography (CT) imaging was performed at baseline, after two cycles, after four cycles, and at the end of the treatment. In addition, CT imaging was repeated every 2 months for 2 years or until the disease had progressed or relapsed. PET‐CT imaging was recommended, but not compulsory.

Complete response was defined as no evidence of disease or disease‐related symptoms. Partial response (PR) was defined as a ≥50% decrease in the sum of the product of the diameters of the masses and no new lesions. Stable disease (SD) was defined failure to attain CR or PR, but not fulfilling the criteria for PD. PD was defined as the appearance of new sites or ≥50% increase in the sum of the product of the diameter of previous lesions from the nadir. The ORR was defined as the proportion of CR and PR patients. DDC was defined as the time interval between the first assessment of CR, PR, and SD, and the first assessment of PD or death from any cause. DOR was defined as the interval between the first assessment of CR or PR and PD or death from any cause. PFS was defined as the time from the first day of the regimen to the documentation of disease progression or death. OS was defined as the time interval from the first day of the regimen to death or the final follow‐up. Treatment efficacy was assessed using the revised Cheson Standard Response.^15^

All AEs were reported from cycle 1, day 1 until 30 days after the last dose of the study drug, regardless of the relationship to treatment. AEs were graded according to the National Cancer Institute Common Terminology Criteria for Adverse Events, Version 5.0.

## 3 RESULTS

### 3.1 Patients characteristics

Fifty‐six patients with evaluable efficacy were included in this study, most of whom were unable to undergo high‐dose chemotherapy or hematopoietic stem cell transplantation due to disease progression, comorbidities, poor response to previous treatments, or previous transplantation failure. There were no significant differences in patient characteristics between the decitabine‐RDHAP group (35 cases) and the RDHAP group (21 cases). Overall, 54% of the patients (*n* = 30) were male, with a male‐to‐female ratio of 1.15:1.00 and median age was 51 years (range 14–70 years). In addition, 73% (*n* = 41) of the patients had stage III–IV disease. The mean number of chemotherapy cycles in the decitabine‐RDHAP and RDHAP groups were 2.83 and 2.38, Two patients in the decitabine‐RDHAP group and two patients in the RDHAP group were double‐hit DLBCL. Four patients in the decitabine‐RDHAP group and one patient in the RDHAP group had an ECOG score of 3 (Table 1).

**TABLE 1:** Patient characteristics

Characteristics	Decitabine‐RDHAP group (*n* = 35)	RDHAP group (*n* = 21)	*p*
Age			0.367
>50 years	21 (60%)	10 (48%)	
Sex			0.128
Male	16 (45%)	14 (67%)	
Stage			0.139
I–II	7 (20%)	8 (38%)	
IPI score			0.333
0–2	17 (49%)	13 (62%)	
Elevated serum LDH	17 (49%)	14 (67%)	0.187.
Elevated β_2_‐MG level	14 (40%)	11 (52%)	0.367
B symptoms present	13 (37%)	9 (43%)	0.672.
Hans somatotype			0.184
Germinal center B‐cell‐lik**e**	9 (26%)	9 (43%)	
Bone marrow invasion	12 (34%)	3 (14%)	0.850
Disease status			0.534
Relapsed type	18 (51%)	8 (38%)	
Refractory type	17 (49%)	13 (62%)	
Number of previous treatment lines			0.213
=1 times	14 (40%)	12 (57%)	
>1 times	21 (60%)	9 (43%)	
Double‐expression DLBCL	17 (49%)	7 (33%)	0.265
Double‐hit DLBCL	2 (6%)	2 (10%)	1.000
Chemotherapy cycle			0.112
≤2	14 (40%)	13 (62%)	
>2	21 (60%)	8 (38%)	
Previous rituximab treatment			1.000
Yes	32 (91%)	20 (95%)	
No	3 (9%)	1 (5%)	
ECOG score			0.367
0–1	14 (40%)	11 (52%)	
2–3	21 (60%)	10 (48%)	
4–5	‐	‐	
Vital organ insufficiency			0.904
Yes	3 (9%)	2 (10%)	

*Note*: Low LDH <245 U/L, high LDH ≥245 U/L; low β2‐MG <3 mg/L, high β2‐MG ≥3 mg/L.

Abbreviations: ECOG, Eastern Cooperative Oncology Group; IPI, International Prognostic Index; LDH, lactate dehydrogenase; RDHAP, rituximab, cisplatin, cytarabine, dexamethasone; β2‐MG, β2‐microglobulin.

### 3.2 Survival

In this study, 10 (29%) patients in the decitabine‐RDHAP group and 4 (19%) patients in the RDHAP group achieved CR. The ORR was 40% in the decitabine‐RDHAP group and 33% in the RDHAP group with no significant difference in the response rate between the two groups (*p* = 0.849) (Table 2).

**TABLE 2:** Response rates of decitabine‐RDHAP and RDHAP regimen

Response	ORR	SD	PD	*p*
Decitabine‐RDHAP group (*n* = 35)	14 (40%)	7 (20%)	14 (40%)	0.849
RDHAP group (*n* = 21)	7 (33%)	6 (29%)	8 (38%)	

Abbreviations: ORR, objective response rate; PD, progressive disease; RDHAP, rituximab, cisplatin, cytarabine, dexamethasone; SD, stable disease.

**FIGURE 1:**
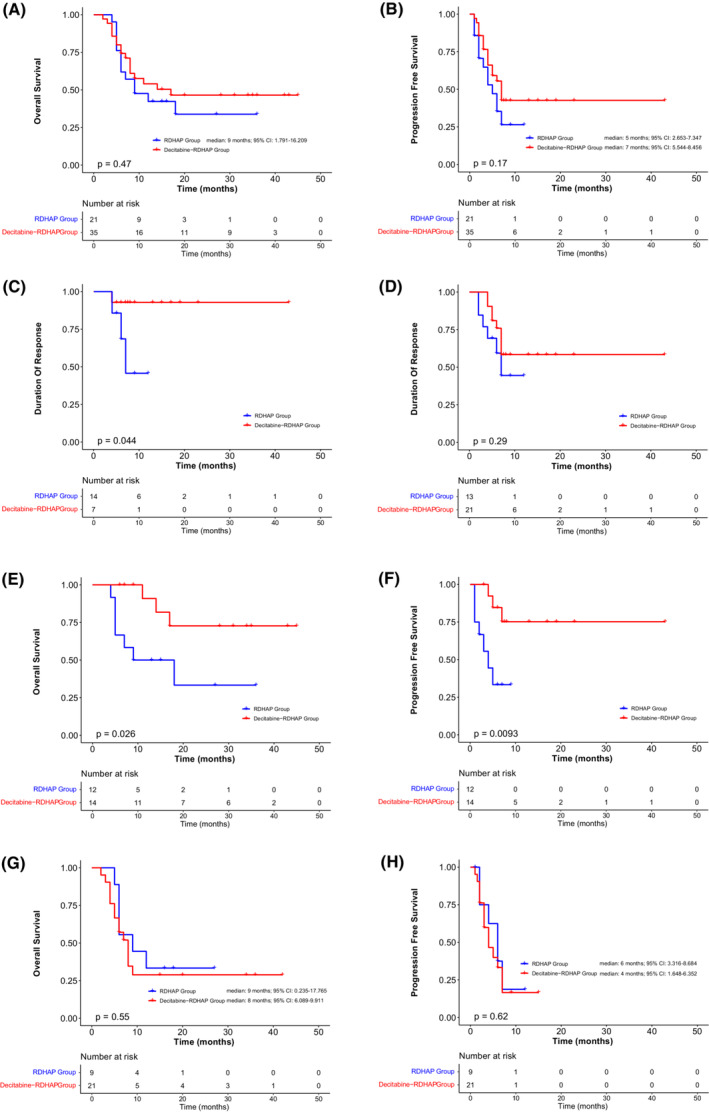
(A, B) OS and PFS for all patients. There were no significant differences between the decitabine‐RDHAP group and RDHAP group (*p* = 0.47, *p* = 0.17, respectively). (C) DOR for in the two treatment arms. The decitabine‐RDHAP group had a better DOR than the RDHAP group (*p* = 0.044). (D) DDC for the two treatment arms. There was no significant difference between decitabine‐RDHAP group and RDHAP group (*p* = 0.29). (E, F) OS and PFS of patients with only one previous line of therapy. The decitabine‐RDHAP group had better OS and PFS than the RDHAP group (*p* = 0.026 and *p* = 0.0093, respectively). (G, H) OS and PFS of patients with more than one previous line of therapy. There were no significant differences in OS and PFS between the two treatment arms (*p* = 0.55, *p* = 0.62, respectively). DDC, duration of disease control; DOR, duration of response; OS, overall survival; PFS, progression‐free survival; RDHAP, rituximab, cisplatin, cytarabine, dexamethasone.

At a median follow‐up of 12 months (hazard ratio [HR]: 4.109 (3.947–20.053)), the median PFS in the decitabine‐RDHAP and RDHAP groups was 7.0 months (95% confidence interval [CI]: 5.544–8.456) and 5.0 months (95% CI: 2.653–7.347) (Figure 1B), respectively; the median OS was 17.0 and 9 months (95% CI: 1.791–16.209) (Figure 1A), respectively. No statistically significant differences in OS, PFS, or DDC were observed between the two groups (*p =* 0.47, *p =* 0.17, and *p =* 0.29, respectively). However, a significant difference in the DOR was observed (*p* = 0.044). For patients with only one previous line, the median PFS in the decitabine‐RDHAP group and RDHAP group was 7 and 4 months, respectively, and the median OS was 17.0 and 13.5 months, respectively, and these differences were significant (*p =* 0.026 and *p =* 0.0093, respectively). The 1‐year OS rates in the decitabine‐RDHAP and RDHAP groups were 54% and 43%, respectively, and the 1‐year PFS rates were 43% and 26%, respectively.

### 3.3 Subsequent treatment

In the decitabine‐RDHAP group, two patients underwent transplantation, one of whom was treated sequentially with chimeric antigen receptor T‐cell (CAR‐T) therapy and had a median OS of 38.5 months; five patients underwent CAR‐T therapy later and two of those patients survived. In the RDHAP group, three patients underwent transplantation and two of these patients survived. However, some patients, because of economic factors or because they did not meet the criteria of CAR‐T therapy, transplantation or other clinical trials, chose maintenance therapy such as lenalidomide (2.86% vs. 14.3%), ibrutinib (8.6% vs. 0%), venetoclax (2.9% vs. 0%), or ibrutinib plus lenalidomide (5.7% vs. 4.8%). However, in patients with SD or PD, some patients with better constitution who could tolerate chemotherapy chose R‐GDP (5.7% vs. 9.5%), R‐Gemox (11.4% vs. 9.5%), R‐EPOCH (5.7% vs. 23.8%), R‐DICE (5.7% vs. 0%), and R‐ESHAP (5.7% vs. 0%), while other patients chose observation or discontinued treatment without drug therapy (25.7% vs. 23.8%). Among all patients achieving SD or PD, eight patients in the decitabine‐RDHAP group and four patients in the RDHAP group responded to subsequent treatment.

At the last follow‐up, 17 deaths occurred in the decitabine‐RDHAP group (four from PD, four from multiple system organ failure, and nine from infection or other nonneoplastic diseases). Thirteen deaths occurred in the RDHAP group (three from PD, five from multiple system organ failure, and five from infection or other nonneoplastic diseases).

### 3.4 Patients subgroups analysis

**FIGURE 2:**
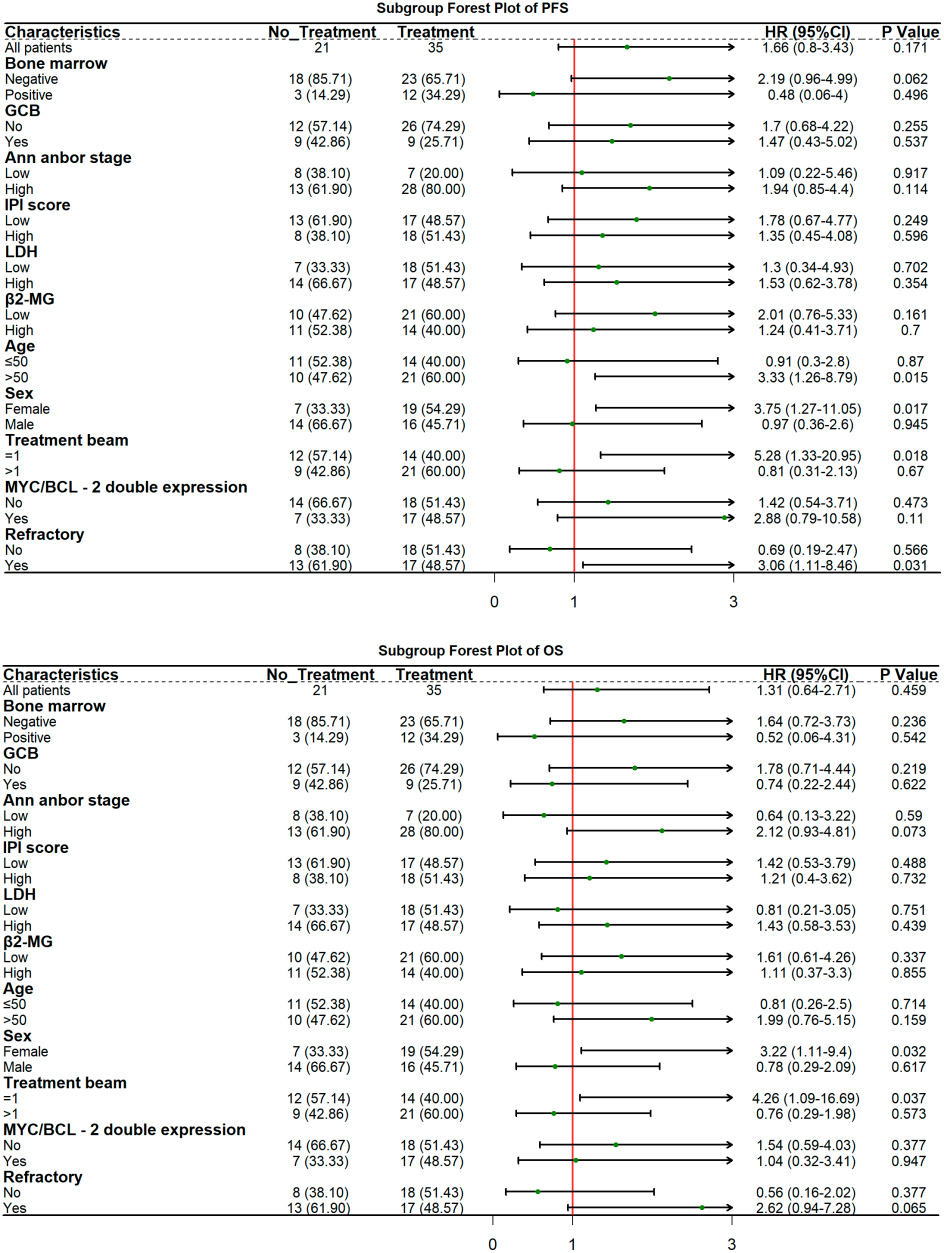
GCB, germinal center B‐cell‐like lymphoma; HR, hazard ratio; IPI, International Prognostic Index; IPI score: low: 0–2, high: 3–5; LDH, lactate dehydrogenase, low LDH <245 U/L, high LDH ≥245 U/L; β2‐MG, β2 microglobulin, low β2‐MG <3 mg/L, high β2‐MG ≥3 mg/L. No treatment: rituximab, cisplatin, cytarabine, dexamethasone. Treatment: decitabine, rituximab, cisplatin, cytarabine, and dexamethasone.

The PFS and OS of the clinical subgroups were analyzed, including bone marrow invasion, cell source, stage, International Prognostic Index (IPI) score, lactate dehydrogenase (LDH) level, β2 microglobulin (β2‐MG) level, age, sex, previous treatment line number, double expression, and refractory status. There were significant differences in PFS between the decitabine‐RDHAP and the RDHAP groups for the following subgroups of patients: female patients, refractory cases, patients aged >50 years, and patients with one previous line of therapy [*p* < 0.05; HR 3.75 (1.27–11.05), HR 3.06 (1.11–8.46), HR 3.33 (1.26–8.79), and HR 5.28 (1.33–20.95), respectively]. OS in the decitabine‐RDHAP group was better than that in the RDHAP group for female patients and patients with only one previous line of therapy (*p* < 0.05), with HRs of 3.22 (1.11–9.40) and 4.26 (1.09–16.69) (Figure 2).

### 3.5 Adverse events

There were no significant differences in the digestive tract, liver, kidney, heart, or neurotoxicity between the two groups. All patients experienced AEs during treatment, most of which were hematological AEs. The most common hematological toxicities included leukopenia (86%), thrombocytopenia (74%), and anemia (57%). In the decitabine‐RDHAP group, five patients (14%) received at least one red blood cell infusion and 11 patients (31%) received at least one platelet infusion, In the RDHAP group, three patients (14%) received at least one red blood cell infusion and two patients (10%) received at least one platelet infusion. In both groups, non‐hematologic toxicity included pneumonia, nausea, numbness in the extremities, liver damage, and insomnia. These symptoms were effectively relieved with appropriate treatment, and there were no fatal AEs.

In the decitabine‐RDHAP group, nine patients had a 20% reduction (cisplatin and cytarabine) due to severe grade 3–4 AEs that did not recover within 2 weeks: six because of a severe decrease in platelet and white blood cell counts; one due to renal toxicity, one due to severe pneumonia, and one due to hepatic toxicity, four of whom delayed the next cycle of chemotherapy.

## 4 DISCUSSION

The primary analysis of the study, with a follow‐up of at least 44 months, showed that decitabine in combination with the RDHAP regimen resulted in a 40% in ORR, an OS of 17 months, and a PFS of 7 months (Figure 1; Table 2). The results were better than those of the RDHAP group, which is similar to the median survival (OS) of 6 months reported in other studies.^16^, ^17^ However, contrary to the results that were anticipated, the differences between the two groups were not significant (Figure 1A,B). This may be explained by the fact that 80% and 61% of the patients in the decitabine‐RDHAP group and the RDHAP group, respectively, had stage III–IV disease with multiple tumor metastases (Table 1). In addition, the epigenetic therapeutic effects of decitabine are S‐phase dependent, and each cycle of therapy can only affect the fraction of the malignancy that enters S‐phase in a small window of time.^18^ In the course of myelodysplastic syndromes treatment, the best response to decitabine can occur after as many as 12 cycles of therapy. We suspect that there can be no significant therapeutic benefit, if DNA methyltransferase is not depleted.^19^ More evidence is required to confirm the efficacy of this strategy. Nevertheless, we consider that this combination has great potential and warrants further investigation in R/R‐DLBCL.

Decitabine, an epigenetic drug, has emerged as a promising treatment option for lymphoma.^20^, ^21^ It may potentiate the actions of other chemotherapy and immunotherapy agents, and may induce long‐lasting responses through priming of the immune system.^22^ In this study, we found that patients who achieved CR or PR had a longer DOR in the decitabine‐RDHAP group. Similar findings were reported in a prospective study of decitabine in relapsed or refractory Hodgkin's lymphoma.^10^ We postulate that decitabine and cytarabine induce cellular reprogramming, which can lead to complete cytogenetic remission in patients and extend the duration of remission.^18^, ^23^ This study found that as a second‐line salvage therapy, decitabine combined with RDHAP group had a better prognosis (Figure 1E–H), and the ORR in the subgroup with one previous treatment accounted for 79% of the decitabine‐RDHAP group (57% in the RDHAP group). A phase II trial of acute myelogenous leukemia reported that decitabine was effective when used early in the treatment.^24^ In a clinical trial of treatment‐naive DLBCL, patients were treated with escalating doses of decitabine before undergoing treatment with R‐CHOP and 86% of patients responded, with a median follow‐up of 12 months, and 71% of patients remained in remission.^25^ These trials focused on treatment‐naive patients, demonstrating the role of decitabine even in the early stages of newly diagnosed tumors. In view of the small number of patients with R/R‐DLBCL receiving decitabine‐RDHAP regimen as second‐line treatment in our study (14 patients in the decitabine‐RDHAP group), caution should be exercised when interpreting these results. We consider that the decitabine‐RDHAP regimen is a promising treatment option for the early treatment of DLBCL.

At the last follow‐up, 64.2% (9/14) of the patients in the decitabine‐RDHAP group had progressed or relapsed, indicating that the long‐term disease control rate of this regimen was limited. Therefore, patients should actively seek alternative treatment to achieve optimal survival. Seven patients in the decitabine‐RDHAP group underwent transplantation or CAR‐T therapy, and four achieved long‐term survival. Among patients with SD or PD, some patients with better constitution chose chemotherapy to control disease progression. In addition, eight patients in the decitabine‐RDHAP group responded to subsequent therapy, which could be associated with the delayed chemosensitization effect of decitabine.^26^ In our study, patients were actively encouraged to undergo transplantation for cure. However, only 18% of patients (10/56) underwent transplantation or CAR‐T therapy after treatment. We analyze the reasons for this situation. Among the patients included in our study, 54% had undergone at least two previous lines of therapy, 55% had an Eastern Cooperative Oncology Group score of 2–3, 73% were in the advanced stages, and some patients did not proceed with transplantation or CAR‐T because of financial circumstances or complications such as infection, cardiac insufficiency, etc. In short, patients were in poor physical condition and could not tolerate HDT‐ASCT or allogeneic hematopoietic stem cell transplantation. Furthermore, these patients have a high tumor burden and are at high risk for continued progression. For these people, decitabine‐RDHAP regimen can be used as bridging therapy followed by transplantation or CAR‐T therapy for better survival.

In subgroup analysis, the decitabine‐RDHAP group showed a greater PFS benefit among patients who were female, with refractory disease, aged >50 years, or had one previous line of therapy treatment. Among patients who were female or had undergone one previous line of therapy treatment, the decitabine‐RDHAP group showed a greater OS benefit (Figure 2). This may be explained by the slow degradation rate of rituximab and decitabine in female patients and the high methylation rate before and after treatment.^14^, ^27^ Although one of the IPI scoring system is the age of >60 years of the patients, some studies drawn different conclusions regarding the relationship between age and prognosis.^28^, ^29^ Perhaps for Chinese patients, aged >50 years have indicated a poor prognosis, which may be related to the diet and physique of Chinese people. We found the frequencies of epigenetic mutations such as DNMT3A, TET2, ASXL1, and IDH2 were higher in older patients.^30^ Hypomethylating drugs such as decitabine are pyrimidine nucleoside analogs that can result in the hypomethylation of DNA and restoration of expression of tumor‐suppressor genes by inhibiting DNA methyltransferases.^31^ Importantly, the decitabine‐RDHAP regimen can benefit refractory patients. Decitabine, an epigenetic drug, can induce reprogramming of chemorefractory DLBCL cell lines, decrease DNA methylation, and augment CTR1 expression to overcome chemotherapy resistance and improve patient outcomes.^14^ It has also been used in B cell lymphoma treatment as a means of improving responsiveness to chemotherapy and overcoming treatment resistance in advanced settings.^13^, ^26^ Interestingly, two double‐hit patients responded to the decitabine‐RDHAP regimen, while two double‐hit patients in the RDHAP group progressed. More intensive therapy may improve survival for MYC‐rearranged lymphoma. However, intensive therapy has not been proven effective in patients with double‐hit lymphoma.^32^ However, the specific research mechanism needs to be explored further.

This study revealed no deaths associated with decitabine treatment. The most common hematological toxicities were thrombocytopenia (74% of patients), leukopenia (86%), and anemia (57%) (Table 3). As a result, in the decitabine‐RDHAP group, nine patients had a 20% reduction in chemotherapy dose (cisplatin and cytarabine), four of whom had delayed treatment. Considering the curative effect, one study suggests that regular treatment should be initiated promptly.^33^ Furthermore, the most common non‐hematologic toxicities included pneumonia, nausea, numbness in the extremities, liver damage, and insomnia, consistent with previous reports.^34^ In this study, the symptoms slowly resolved after supportive treatment.

**TABLE 3:** Adverse events

AEs	Decitabine‐RDHAP (*n* = 35)		RDHAP (*n* = 21)		*p*
	Grade 1–4	Grade 3–4	Grade 1–4	Grade 3‐4	
Leukopenia	30 (86%)	18 (51%)	17 (81%)	6 (29%)	0.925
Thrombocytopenia	26 (74%)	15 (43%)	14 (67%)	5 (24%)	0.541
Anemia	20 (57%)	12 (34%)	14 (67%)	3 (14%)	0.480
Pneumonia	25 (71%)	9 (26%)	10 (48%)	4 (19%)	0.075
Mucositis	10 (29%)	6 (17%)	7 (33%)	3 (14%)	0.708
ALT/AST elevations	20 (57%)	14 (40%)	14 (67%)	4 (19%)	0.480
Renal insufficiency	18 (51%)	10 (29%)	10 (48%)	2 (10%)	0.783
Numbness	25 (71%)	8 (23%)	12 (57%)	4 (19%)	0.274
Diarrhea	5 (14%)	1 (3%)	2 (10%)	1 (5%)	0.917
Nausea	22 (63%)	8 (23%)	14 (67%)	6 (29%)	0.773
Constipation	13 (37%)	6 (17%)	6 (29%)	3 (14%)	0.512
Insomnia	20 (57%)	8 (23%)	10 (48%)	3 (14%)	0.489

Abbreviations: AE, adverse event; ALT, glutamic‐pyruvic transaminase; AST, glutamic oxaloacetic transaminase; RDHAP, rituximab, cisplatin, cytarabine, dexamethasone.

At present, there are some limitations in the study, there are few clinical studies on the treatment of DLBCL with decitabine in China. In addition, many of the patients received other treatment strategies after participating in our study. Besides, our research may be confined to a small sample size. To further study the effect of decitabine combined with RDHAP regimen in DLBCL, more elaborate randomized controlled trial design are needed in subsequent studies.

In conclusion, our study indicates that the decitabine‐RDHAP regimen is effective and well‐tolerated and is a promising salvage regimen for patients with R/R‐DLBCL.

## AUTHOR CONTRIBUTIONS

**Xiaoshuang Kong:** Writing – original draft (equal). **Xudong Zhang:** Formal analysis (equal); funding acquisition (equal); writing – review and editing (equal). **Mengjie Ding:** Software (equal); supervision (equal); validation (equal); visualization (equal). **Xiaoyan Feng:** Data curation (equal); formal analysis (equal); investigation (equal). **Meng Dong:** Data curation (equal); investigation (equal); methodology (equal); visualization (equal). **Lei Zhang:** Conceptualization (equal); supervision (equal). **Xiaorui Fu:** Investigation (equal); software (equal); visualization (equal). **Ling Li:** Data curation (equal); investigation (equal); methodology (equal). **Xin Li:** Supervision (equal); validation (equal); visualization (equal). **Zhenchang Sun:** Formal analysis (equal); investigation (equal); resources (equal); validation (equal). **Jiaqin Yan:** Data curation (equal); formal analysis (equal); software (equal). **Xinhua Wang:** Supervision (equal); validation (equal); visualization (equal). **Xiaolong Wu:** Conceptualization (equal); data curation (equal); formal analysis (equal); investigation (equal). **Qingjiang Chen:** Formal analysis (equal); funding acquisition (equal); investigation (equal); methodology (equal). **Mingzhi Zhang:** Formal analysis (equal); funding acquisition (equal); project administration (equal); resources (equal). **Linan Zhu:** Conceptualization (equal); data curation (equal); project administration (equal); supervision (equal); validation (equal); writing – review and editing (equal).

## FUNDING INFORMATION

National Natural Science Foundation of China, Grant Numbers: 82070210. Medical science and technique key project in Henan Province, Grant Numbers: SBGJ202001008.

## CONFLICT OF INTEREST

All authors declare no conflict of interest.

## CLINICAL TRIAL REGISTRATION NUMBER

NCT03579082.

## ETHICAL APPROVAL STATEMENT

The study was approved by the Human Ethics Committee of the First Affiliated Hospital of Zhengzhou University and was conducted in accordance with the principles outlined in the Declaration of Helsinki. All the patients or their family members signed an informed consent form.

## Data Availability

Expects data sharing.
